# Glucocorticoid Receptor Regulates *TNFSF11* Transcription by Binding to Glucocorticoid Responsive Element in *TNFSF11* Proximal Promoter Region

**DOI:** 10.3390/ijms22031054

**Published:** 2021-01-21

**Authors:** Nika Lovšin, Janja Marc

**Affiliations:** The Chair of Clinical Biochemistry, Faculty of Pharmacy, University of Ljubljana, Aškerčeva cesta 7, 1000 Ljubljana, Slovenia; janja.marc@ffa.uni-lj.si

**Keywords:** glucocorticoid receptor, NR3C1, RANKL, TNFSF11, glucocorticoid induced osteoporosis

## Abstract

Glucocorticoid osteoporosis is a serious side effect of long term glucocorticoid uptake and it is caused by osteoblast apoptosis and imbalance in the major bone remodeling pathway RANK/RANKL/OPG. The impact of glucocorticoid on the maintenance of RANK/RANKL/OPG is well explored; dexamethasone was shown to disturb the ratio between *OPG* and *RANKL* level by decreasing the expression level of *OPG* and increasing level of *RANKL*. Here, were aimed to decipher whether glucocorticoid receptor directly influences *RANKL* promoter activity and its transcriptional regulation. We demonstrate that overexpression of glucocorticoid receptor (GR) NR3C1 increased *RANKL* promoter activity in human osteosarcoma, cervical cancer (2-fold) and adenocarcinoma cells (4.5-fold). Mutational analysis revealed that +352 site in the *RANKL* promoter is functional glucocorticoid responsive element (GRE) since the effect of GR on *RANKL* promoter activity was diminished by mutation at this site. Overexpression of *NR3C1* upregulated *RANKL* mRNA expression 1.5-fold in human A549 and HOS cells. On the other hand silencing of *NR3C1* caused slight decrease in *RANKL* mRNA level, suggesting that NR3C1 directly accounts for *RANKL* transcriptional regulation. Using electrophoretic mobility shift assay we demonstrate that NR3C1 binds to the proximal *RANKL* promoter region. Our study provides evidences that NR3C1 directly upregulates *RANKL* transcription in human cell lines and connects the missing link in the mechanism of RANK/RANKL/OPG imbalance of glucocorticoid induced osteoporosis.

## 1. Introduction

The Glucocorticoid induced osteoporosis (GIO) is the most frequent cause of secondary osteoporosis [1,2,3]. Patients treated with glucocorticoids have increased risk for bone fracture and increased bone resorption [4,5,6]. Glucocorticoid-induced bone loss is primarily caused by apoptosis of osteoblast and secondarily by increase in bone resorption. Increased bone resorption at the early phase of GIO is attributed with increased osteoclast formation and activity of mature osteoclasts [7,8]. At the later stage of GIO, long term bone loss is associated with decrease in bone formation [9,10]. Osteoclasts are monocyte derived multinuclear cells responsible for bone absorption. Osteoclast are formed through osteoclastogenesis induced by osteoblast secreted receptor activator of nuclear factor κ B ligand (RANKL), which is a key signaling molecule that induces differentiation of bone macrophages into mature osteoclasts, and their activation. RANKL induces osteoclastogenesis by binding to RANK receptor on osteoclast precursor cells. Tight balance between osteoclast and osteoblast formation and degradation prerequisites proper bone formation and resorption processes. The key system regulating those bone remodelling processes is a RANK/RANKL/OPG pathway. Upregulation of *RANKL* in osteoblasts, increases RANKL secretion that activates osteoclasts formation and an increase in bone resorption [11]. Osteoprotegerin (OPG) is a soluble decoy receptor for RANKL and prevents its interactions with RANK, thus inhibits osteoclastogenesis and bone resorption. In osteoporosis, *RANKL* expression in bone tissues is upregulated, which leads to stronger bone resorption and decrease in bone mineral density [12].

RANKL belongs to the tumour necrosis factor (TNF) family of cytokines, and is encoded by *TNFSF11* gene. It is found as membrane-bound and soluble forms, the latter of which is produced by alternative splicing or by proteolytic cleavage of membrane-bound RANKL [13,14,15]. RANKL is involved in numerous physiological processes as well as bone metabolism, such as in T-cell functions, and lymph node and mammary gland development [16,17,18,19]. *RANKL* is expressed in cells involved in bone formation (e.g., osteoblasts, their precursors, and osteocytes), immune cells, malignant cells, and other cell types [20]. RANKL is also involved in pathological processes, such as migration of tumor cells and development of bone metastases in diseases like breast, lung and prostate cancers [21,22,23,24,25]. *RANKL* expression is regulated by parathyroid hormone (PTH), vitamin 1,25(OH)2D3, Runt-related transcription factor 2 (Runx2), interleukin (IL)-6-type cytokines [26,27], molecules of the Wnt/β-catenin signaling pathway [28,29,30,31,32,33,34,35], and c-Fos in activated T cells that act through proximal or/and distal promoter regions [20]. We demonstrated that *RANKL* gene expression is also mediated by gender specific transcription factor SRY [12].

Glucocorticoid receptor (GR) has been found in most of the human cells and can regulate transcription of hundreds of genes depending on the pathophysiological state of cell [36,37]. GR is encoded by *NR3C1* gene, which is transcribed into two isoforms, GR-α and GR-β that modulate transcriptional regulation either directly or through transactivation with other transcription factors [1,38]. When glucocorticoid (GC) (e.g., dexamethasone) binds GR, causes its activation, and translocation into the nucleus. In the nucleus, GR transactivates or represses promoters with glucocorticoid response elements (GRE) [39]. Besides direct binding to GREs, GR can modulate gene expression by binding to other transcription factors in the process named tethering [38,40].

Glucocorticoid dexamethasone was shown to cause imbalance in RANK/RANKL/OPG pathway and bone resorption by reduction of osteoprotegerin (OPG) level and also by modifying *RANKL* level [39,41,42]. The impact of dexamethasone on *RANKL* expression varies between the cell types depending on their nature of origin [39]. In calvaria, dexamethasone upregulates *OPG* [7]. In human osteoblastic cell lines, dexamethasone downregulated *OPG* and upregulated *RANKL* [8,43]. The level of *OPG* and *RANKL* expression is influenced by time and concentration of dexamethasone [8]. In human primary osteoblasts, dexamethasone was shown to decrease *OPG* and increase *RANKL* expression level [44,45]. Dexamethasone itself also caused slight increase in *RANKL* gene expression in bone-marrow-derived osteoblastic/stromal cell line ST2, whereas *OPG* was strongly downregulated in this cell line [41]. In mice treated with GC, bone loss and osteoclast formation was prevented by treatment with denosumab, anti-RANKL antibodies, suggesting direct impact of GC on RANKL induced osteoclastogenesis [46]. Kitazawa et al. has identified dexamethasone and vitamin D3 responsive elements in the mouse proximal *RANKL* promoter region suggesting that GC can directly impact *RANKL* transcriptional regulation [41,47]. The authors suggested that −642/−628 putative GRE half sites (TTTCCTGACTGTTCC) or AP-1 binding site are dexamethasone responsive sites [41]. A strong synergistic effect of vitD3 and dexamethasone suggested that VDR-RXRb heterodimer is capable of forming more durable/effective complexes with cofactors in the presence of GR [47]. Moreover, a recent mouse model study revealed that monomeric GR suffice for upregulation of *RANKL* expression in osteoblasts implying that GC upregulate *RANKL* expression through monomeric GR [48]. All these data demonstrate that *RANKL* expression is influenced by GCs that act through GR pathway. Whether GR activates *RANKL* promoter directly by DNA binding or by tethering with other transcription factors remains unknown.

Here, we explored whether GR directly upregulates human *RANKL* expression. We demonstrate that GR increases human *RANKL* promoter activity in human A549, HeLa and HOS cells via at least two GRE sites in the human *RANKL* promotor region; −642, and +352 relative to transcription start site (TSS). We provide an evidence that GR activates *RANKL* promoter through GRE site at +352, since the mutated promoter was not affected by overexpression of GR. This is the first study demonstrating that GR can directly regulate human *RANKL* gene by binding to its proximal promoter region. We show that GR caused upregulation of *RANKL* in human adenocarcinoma A549 and human osteosarcoma HOS cells. Altogether, our results indicate that GR directly activates human *RANKL* promoter and regulates *RANKL* expression.

## 2. Results

### 2.1. Overexpressed Glucocorticoid Receptor NR3C1 Increases RANKL Promoter Activity in A549, HOS and HeLa Cells

**Figure 1:**
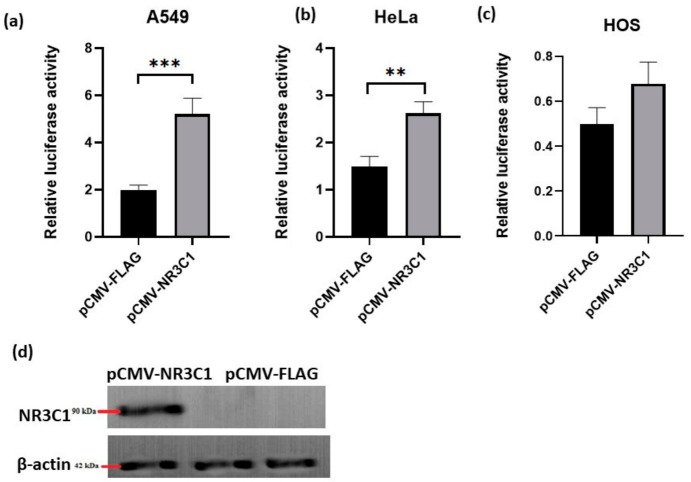
Overexpression of gluccocorticoid receptor causes activation of *RANKL* promoter in A549, HeLa and HOS cells. (**a**) A549, cells were co-transfected with the luciferase reporter constructs harboring *RANKL* promoter region 889 bp, (pGL3-RANKL100), and 24 h after transfection luciferase activity was measured. Relative luciferase activity is shown and it was calculated by normalization of Firefly luciferase activity to Renilla luciferase activity. (**b**) HeLa cells were co-transfected with the luciferase reporter constructs harboring *RANKL* promoter region 889 bp, (pGL3-RANKL100), and 24 h after transfection luciferase activity was measured. (**c**) HOS cells cells were co-transfected with the luciferase reporter constructs harboring *RANKL* promoter region 889 bp, (pGL3-RANKL100), and 24 h after transfection luciferase activity was measured. (**d**) Expression of overexpressed protein was confirmed by Western blot using anti-FLAG antibodies. *β*-actin was used as a loading control. All the experiments were performed at least three times independently in parallels. The results are expressed as the mean ±SEM. **–*p* < 0.01, ***–*p* < 0.001.

Here, were aimed to examine whether glucocorticoid receptor directly influences *RANKL* promoter activity and its transcriptional regulation. Since the expression of *RANKL* gene plays a crucial role in bone remodelling processes and it is modulated in various cancers [49], three different human cell lines were included in the experiments, human lung cancer cells (A549), human osteosarcoma cells (HOS) and human cervical cancer cells (HeLa). To examine the impact of glucocorticoid receptor NR3C1 on the activity of *RANKL* promoter, cells were co-transfected with plasmids encoding glucocorticoid receptor NR3C1 (pCMV-NR3C1) and pGL3 plasmid with 898 bp *RANKL* proximal promoter region (plasmid pGL3-RANKL-100 (region −789 to +100 relative to transcription start site [12]). Promoter activity was measured by luciferase assays 24 h after transfection. In A549 cells, in the presence of overexpressed NR3C1 promoter activity of pGL3-RANKL-100 increased 2.6 fold (*p* = 0.0008) in comparison with an empty vector (Figure 1a). In HeLa cells, overexpression of NR3C1 caused 1.7 fold (*p* = 0.0063) increase in promoter activity in comparison with an empty vector (Figure 1b). In HOS cells, overexpression of *NR3C1* increased the promoter activity when comparing to an empty vector, yet the change was not significant (Figure 1c). Overexpression of glucocorticoid receptor NR3C1 protein was verified by immunoblot (Figure 1d). Increase in *RANKL* promoter activity in the presence of NR3C1 suggests that *RANKL* promoter is directly inducible by glucocorticoid receptor.

**Figure 2:**
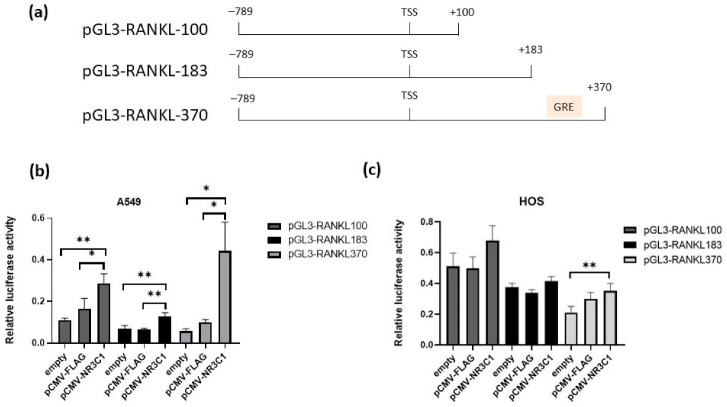
Glucocorticoid receptor NR3C1 potently increases *RANKL* promoter activity of the region including a novel Glucocorticoid Responsive Element (GRE) at +352 site. (**a**) Schematic representation of the human *RANKL* promoter region employed in the luciferase assays. Region from −789 to +370 relative to the transcription start site (TSS) and two length deletions were employed in the study. A novel glucocorticoid response element (GRE) at the site +352 is depicted pink in construct pGL3-RANKL-370. (**b**) A549 cells were co-transfected with the luciferase reporter constructs harboring three *RANKL* promoter regions (pGL3-RANKL100, pGL3-RANKL183, pGL3-RANKL370) and 24 h after transfection luciferase activity was measured. Relative luciferase activity is shown and it was calculated by normalization of Firefly luciferase activity to Renilla luciferase activity. (**c**) HOS cells were co-transfected with the luciferase reporter constructs harboring three *RANKL* promoter regions (pGL3-RANKL100, pGL3-RANKL183, pGL3-RANKL370) and 24 h after transfection luciferase activity was measured. Relative luciferase activity is shown and it was calculated by normalization of Firefly luciferase activity to Renilla luciferase activity. All the experiments were performed at least three times independently in parallels. The results are expressed as the mean ±SEM. *–*p* < 0.05, **–*p* < 0.01.

In bioinformatics analysis, we identified GRE site in the *RANKL* exon 1 region +342\+352 relative to transcription start site (Figure 2a). Next, we prepared plasmids harbouring different lengths of *RANKL* promoter region, also plasmid including newly identified GRE site (plasmids pGL3-RANKL-183, pGL3-RANKL-370) (Figure 2a). A549 and HOS cells were transfected with promoter length mutations (plasmids pGL3-RANKL-100 or pGL3-RANKL-183 or pGL3-RANKL-370) and pCMV-NR3C1 or an empty plasmid (pCMV-FLAG) and the luciferase activity was measured 24 h after transfection. Again, in A549 cells, overexpression of NR3C1 caused significant increase in promoter activity for all three lengths of the *RANKL* promoter. Promoter activity of plasmid pGL3-RANKL-100 (region −789 to +100 relative to TSS) increased 1.7 fold in comparison with an empty vector (*p* = 0.0376), pGL3-RANKL-183 (region −789 to +183 relative to TSS) 1.9 fold (*p* = 0.0024) and plasmid pGL3-RANKL-370 (region −789 to +370 relative to TSS) which includes novel GRE site 4.5 fold (*p* = 0.0224) in comparison with an empty vector (Figure 2b). Promoter activity of the *RANKL* promoter region that includes putative GRE site at +352 (pGL3-RANKL370) was 2.3-fold higher than that of a shorter promoter region (−789 to +183) (pGL3RANKL-183), suggesting that putative GRE site at +352 is a functional GRE site and that overexpression of GR increases *RANKL* promoter activity by binding to this site. Promoter activity of *RANKL* promoter length mutations also increased in the presence of GR in HOS cells, yet changes were not significant (Figure 2c).

### 2.2. Mutation in Glucocorticoid Responsive Element Alleviates Effect of Glucocorticoid Receptor on RANKL Promoter Activity

**Figure 3:**
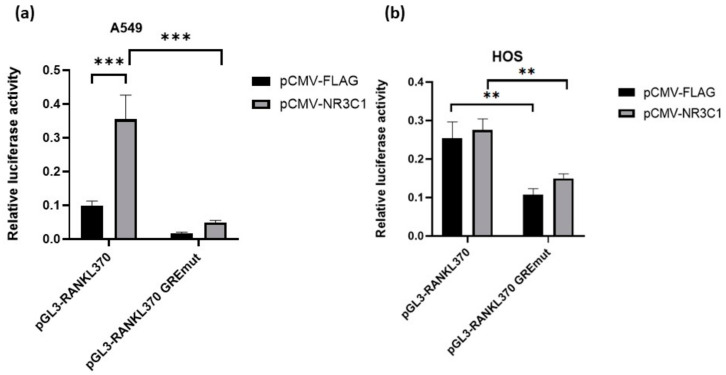
Mutation in Glucocorticoid Responsive Element alleviates effect of GR on *RANKL* promoter activity in A549 cells. (**a**,**b**) A549 and HOS cells were co-transfected with the luciferase reporter constructs harbouring *RANKL* promoter region pGL3-RANKL370 or plasmid with introduced mutation at GRE +352 site (pGL3-RANKL370 GREmut) and pCMV-NR3C1 or empty plasmid (pCMV-FLAG) and 24 h after transfection luciferase activity was measured. Relative luciferase activity is shown and it was calculated by normalization of Firefly luciferase activity to Renilla luciferase activity. All the experiments were performed at least three times independently in parallels. The results are expressed as the mean ±SEM. **–*p* < 0.01, ***–*p* < 0.001.

Next, to examine whether GRE site directly influences the *RANKL* promoter activity, we introduced mutation in the putative GRE site (+352 relative to TSS) (pGL3-RANKL370 GREmut plasmid) and performed luciferase assays in A549 and HOS cells. Overexpression of NR3C1 did not cause significant change in promoter activity of pGL3-RANKL370-GREmut in comparison with an empty vector in any of the tested cells confirming that GRE at +352 is important for induction of *RANKL* promoter activity by NR3C1. In fact, introduction of mutation at GRE site caused 7.9-fold (*p* < 0.0001) and 1.8-fold (*p* = 0.009) lower promoter activity in the presence of GR in A549 and HOS cells, respectively (Figure 3). Interestingly, even in the absence of overexpressed NR3C1, wild type promoter activity was 5-fold and 2.3-fold higher than in the GRE mutated promoter in both cell types in A549 and HOS cells, respectively. Higher promoter activity of the wild type promoter in comparison with the GREmut sequence in the absence of overexpressed NR3C1 implies that GRE, at +352 site, is important for regulation of *RANKL* promoter activity and that endogenous transcription factors contribute to activation of *RANKL* promoter in this region. To summarize, our results indicate that GRE site (+352 relative to TSS) is important for GR activation of *RANKL* transcription in HOS and A549 cells. Moreover, endogenous transcription factors are responsible for activation of *RANKL* promoter (in the region +183 to +370), and this effect is potentiated by overexpressed GR.

### 2.3. Glucocorticoid Receptor Binds to the RANKL Proximal Promoter Region

**Figure 4:**
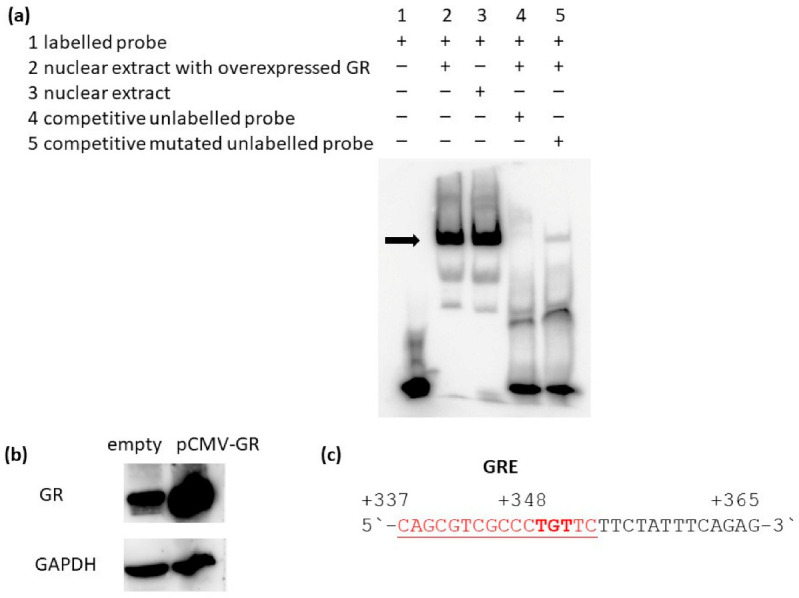
GR directly binds to the putative GRE site at +352 of the human *RANKL* proximal promoter. (**a**) Electrophoretic mobility shift assays using 293T nuclear extracts and biotinylated oligonucleotide probes containing site +352 downstream of the *RANKL* transcription start. Lane 1, negative control without nuclear extract; lane 2, nuclear extract with overexpressed GR protein added; lane 3, nuclear extract from untransfected cells, lane 4, competitive unlabelled probes added; lane 5, mutated competitive unlabelled probe added. (**b**) Western blotting using anti-GR antibodies of cell lysates with transfected (GR) and untransfected nuclear extracts (empty) used for the electrophoretic mobility shift assays in (**a**). anti-GAPDH was used as a loading control. (**c**) Nucleotide sequence of the biotinylated probe, GRE site is underlined.

To examine whether GR directly binds to the putative GRE binding site at +352 relative to TSS of the human *RANKL* proximal promoter region, we performed electrophoretic mobility shift assay. For that purpose, HEK293T cells were transfected with pCMV-NR3C1 plasmid and nuclear extracts were isolated. Nuclear extracts with overexpressed GR or prepared from untransfected cells were incubated with biotinylated probe with GRE sequence at position +352. In parallel, incubations with unlabelled competitive probe (the same sequence as biotinylated probe) and with mutated probe (with mutations at the GRE +352 site) were performed. In case a protein binds to the biotinylated probe, a shift on the gel appears. In the presence of nuclear extract from cells with overexpressed GR, a shift of biotinylated oligonucleotides appeared indicating direct binding of a protein from the nuclear extract to the biotinylated probe (Figure 4a, lane 2). The specificity of the binding was confirmed using unlabelled competitive oligonucleotides in 200-fold molar excess, where a shift disappears (Figure 4a, lane 4). When mutated unlabelled competitive oligonucleotide was added in the reaction, a shift reappeared indicating that unlabelled mutated competitive oligonucleotides did not bind to a protein from the nuclear extract (Figure 4a, lane 5). The presence of overexpressed GR protein in the nuclear extract was confirmed by Western blot (Figure 4b). A shift of labelled probe appeared also in untransfected cells, suggesting that endogenous GR binds to the labelled probe. Indeed, in both cell lysates (with overexpressed and empty cells), a bend resembling to endogenous GR appeared (Figure 4b), overexpressed protein with a higher intensity. This suggests that cells contain sufficient amount of endogenous GR to cause supershift in the EMSA assay. Results of the EMSA experiment indicate that GR directly binds to the *RANKL* proximal promoter region at site +352 (Figure 4c).

### 2.4. Overexpression of Glucocorticoid Receptor Upregulates RANKL Expression in A549 and HOS Cells

**Figure 5:**
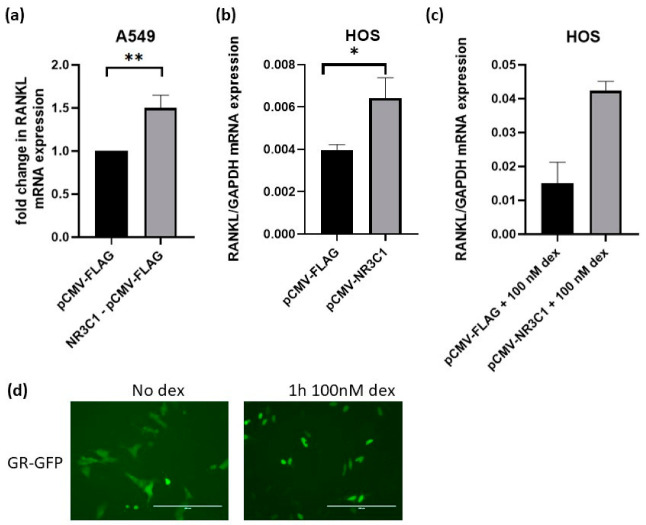
Overexpressed glucocorticoid receptor NR3C1 upregulates expression of *RANKL* in A549 and HOS cells. (**a**,**b**) A549 and HOS cells were transfected with pCMV-NR3C1 or empty plasmid (pCMV-FLAG) and 72 h after transfection RNA was isolated and level of *RANKL* mRNA measured by quantitative PCR. (**c**) HOS cells were transfected with NR3C1 or empty plasmid and treated with 100 nM dexamethasone for 72 h when the level of *RANKL* expression was measured. (**d**) HOS cells were transfected with fluorescently tagged GR (GR-GFP) and 24 h after transfection exposed for 1 h to 100 nM dexamethasone. Translocalisation of GR to the nucleus was observed. All of the data are presented as mean relative *RANKL* expressions after normalization with GAPDH expression. The results are expressed as the mean ±SEM. *–*p* < 0.05, **–*p* < 0.01, Scale bars: 200 µm.

**Figure 6:**
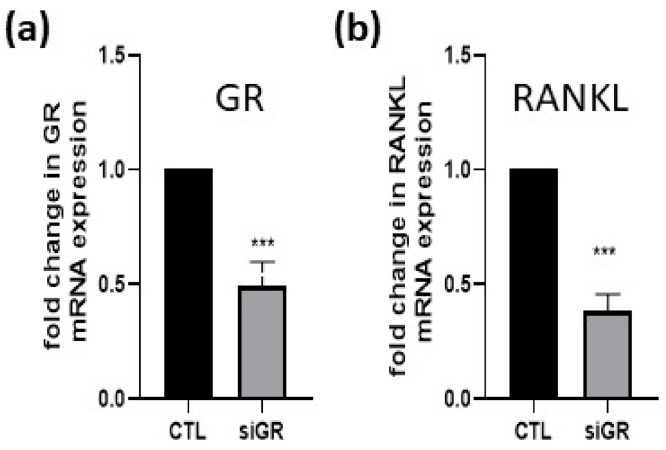
Silencing of *NR3C1* in HOS downregulated *RANKL* expression. (**a**) HOS cells were transfected with siGR or control siRNA (siCTL) and 72 h after transfection RNA was isolated and level of *GR* mRNA measured by quantitative PCR. (**b**) HOS cells were transfected with siGR or control siRNA (siCTL) and 72 h after transfection RNA was isolated and level of *RANKL* mRNA measured by quantitative PCR. All of the data are presented as mean relative gene expressions after normalization with GAPDH expression. Expression in control siCTL samples is set to 1. The results are expressed as the mean ± SEM. ***–*p* < 0.001.

Since, in our *RANKL* promoter activity assays glucocorticoid receptor activated *RANKL* promoter, we next addressed the question whether NR3C1 also increases the mRNA level of *RANKL* in A549 and HOS cells. For that purpose, A549 and HOS cells were overexpressed with pCMV-NR3C1 plasmid and an empty control plasmid and the level of *RANKL* mRNA was measured by qPCR 72 h after transfection. The overexpression of NR3C1 caused 1.5-fold (*p* = 0.0059) and 1.6-fold (*p* = 0.0163) upregulation of *RANKL* in A549 and HOS cells, respectively (Figure 5a,b). We hypothesized that addition of dexamethasone will cause stronger effect of GR overexpression on the level of *RANKL* mRNA. To test this hypothesis, we performed experiment in the presence of dexamethasone. The expression of *RANKL* was measured after treatment of cells with 100 nM glucocorticoid dexamethasone, and the expression of *RANKL* was 2.8 fold higher (*p* = 0.055) in the presence of NR3C1 in comparison with an empty plasmid (Figure 5c). The impact of 100 nM dexamethasone on translocation of GR to the nucleus in shown in Figure 5d. We next hypothesized that silencing of endogenous GR would cause a decrease in *RANKL* expression in HOS cells. To examine whether silencing of endogenous *NR3C1* causes any changes in *RANKL* expression, *NR3C1* was silenced in HOS with siRNA and expression of *RANKL* was measured 72 h after transfection. In HOS cells, 0.49 fold (*p* = 0.0004) change in *NR3C1* level was observed, which caused 0.38 fold change in *RANKL* expression (Figure 6a,b) suggesting that silencing of endogenous GR affects the expression of *RANKL*.

## 3. Discussion

Glucocorticoid osteoporosis is a serious side effect of long term glucocorticoid uptake and it is caused by imbalance in the major bone remodeling pathway RANK/RANL/OPG. The impact of glucocorticoid on the maintenance of RANK/RANL/OPG is well documented; dexamethasone was shown to disturb the ratio between *OPG* and *RANKL* level by downregulation *OPG* and in some case upregulation of *RANKL*. In this study, we examined how GR affects transcriptional regulation of human *RANKL* gene. We show that GR causes an increase in *RANKL* promoter activity and an increase in *RANKL* mRNA expression level. We demonstrate that GR acts through direct binding on GRE site at +352 in the *RANKL* promoter region.

Given that GR affects *RANKL* expression and plays an important role in GIO, we examined a direct effect of GR on the activity of the human *RANKL* promoter. Three regions of the human *RANKL* proximal promoter were analyzed harboring previously reported putative GRE half site (−679; corresponding to mouse −642/−628 site) and putative GRE site at +352 relative to transcription start site. Addition of GR caused higher increase in promoter activity of the region overlapping putative GRE site at +352 in comparison with the region overlapping only putative GRE site at −679. Therefore, our results confirm the results obtained with a mouse *RANKL* promoter [47] and identify a novel putative GRE site at +352 that is involved in the activation of *RANKL* promoter. Moreover, our results demonstrate that GRE residing in the untranslated region of *RANKL* promoter is more important for activation of *RANKL* promotor activity with GR than GRE at −679 site. Namely, +352 region was more inducible by GR than previously reported putative GRE site at −679. Indeed, promoter activity of the region from −789 to +370 in the presence of NR3C1 was higher in comparison with a shorter region from −789 to +100 suggesting that GRE in the untranslated region plays an important role in transcriptional regulation of *RANKL*. Using EMSA, we demonstrate that GR from the nuclear extract directly binds to this +352 region of the *RANKL* promoter confirming the importance of the newly identified GRE in the regulation of *RANKL* gene. In one fifth of the genes that bear GREs, GR binding sites have been reported to reside upstream from transcription start site, yet small portion of GREs have been also identified in the first intron and exon [50], which we also show here for *RANKL* gene. Moreover, several GREs in a single gene have also been identified in other genes, which probably enhances the responsiveness to GC [50]. Our results also corroborated with the results of the ENCODE project, where chromatin immunoprecipitation-sequencing revealed NR3C1 binding to *RANKL* gene in A549 cells treated with 100 nM dexamethasone [51]. Our results indicate that the impact of GR on the *RANKL* expression is very complex. There are at least two regions in the proximal RANKL promoter region that can be directly affected by GR. This provides important knowledge in the understanding the complexity of glucocorticoid induced osteoporosis.

In our experimental setup, in human osteosarcoma and adenocarcinoma cells, addition of GR caused an increase in *RANKL* mRNA level which is similar to previously reported results obtained on dexamethasone treated human primary osteoblast [44] and mouse bone marrow stromal cells treated with dexamethasone and vitamin D3 [41,47]. It was demonstrated that vitamin D3 and dexamethasone synergistically increase expression of *RANKL* in stromal ST2 cells. Dexamethasone was also shown to act synergistically with vitamin D3 to enhance osteoclast formation by increasing *RANKL* mRNA expression in bone marrow stromal primary and transformed cells [48]. Reports on how glucocorticoids affect *RANKL* expression differ depending on the investigated cell types and experimental setups. Comparison between activation of *RANKL* promoter in three different cell types revealed that there is significant difference in transactivation of *RANKL* promoter in the presence of GR between cell systems and that fold of activation differs between cells. Our results indicate that transcription machinery of particular cell system affects *RANKL* promoter activity and corroborates with previous results that GC differently affect *RANKL* expression in different cell types. Besides, different transcriptional machinery, the existence of various mechanism by which GR can transactivate promoters, contribute to those differences.

In vivo relevance of our results was provided by mouse model study, where mice treated with vitamin D3 and dexamethasone showed potentiated osteoclast formation [48]. Treatment of mouse bone marrow stromal cells caused 24-fold increase in *RANKL* expression 6 days after treatment, and decrease in *OPG* mRNA level and was also confirmed at the protein level [48]. The level of endogenous GR was unaffected by dexamethasone and vitamin D3 treatment. However, in mice without GR, combination of dexamethasone and vitamin D3, had no impact on the expression of *RANKL* indicating that dexamethasone and vitamin D3 upregulate *RANKL* transcription via GR transcriptional activation. Interestingly, in mice where dimerization of GR was hindered, dexamethasone and vitamin D3 still caused upregulation of *RANKL* implying that monomeric GR suffice for *RANKL* upregulation and enhanced osteoclastogenesis [48]. Here, we identified GRE site in *RANKL* promoter with one conserved GRE site (CAGCGTCGCCCTGTTCTTC), which mutation alleviates promoter activation, probably due to hindered binding of GR to DNA. Therefore, we propose that one of the mechanisms by which GR upregulates *RANKL* expression is via direct binding of monomeric GR unit to the +352 GRE site of the *RANKL* promoter. Further studies to elucidate whether other transcription factors are involved in this transactivation are required.

In glucocorticoid induced osteoporosis, GC cause bone loss through decrease in osteoblast formation and stimulated osteoclastogenesis. For both processes, balanced ratio in RANK/RANKL/OPG is essential. Here, we focused on the deciphering mechanism behind increase in *RANKL* level. We demonstrate that overexpression of GR caused activation of *RANKL* promoter and an increase in *RANKL* expression in human lung cancer and osteosarcoma cells; probably through direct binding to *RANKL* promoter at the region +352. We provide a link between upregulation of *RANKL* in patients treated with GC and activation of *RANKL* promoter. Our results imply that GC directly influences *RANKL* expression via GR induced activation of *RANKL* transcription.

## 4. Materials and Methods

### 4.1. Plasmid Constructs

Human proximal *RANKL* promoter region (from −798 bp to +370 bp relatively to the transcription start site) was PCR amplified from the human genomic DNA with Q5^®^ High-Fidelity DNA Polymerase (New England Biolabs, Ipswich, MA, USA) using RANKL370 f and RANKL370 rev primers (Supplementary Table S1) and cloned through BglII and HindIII sites into the pGL3 basic vector (Promega, Madison, WI, USA). Cloning resulted in the pGL3-RANKL370 plasmid vector that was sequence verified. Mutations in the GRE region of *RANKL* promoter were introduced with the Quick-Change II Site-Directed Mutagenesis kits (Stratagene, San Diego, CA, USA) using primers listed in the Supplementary Table S1. All the plasmid vectors were sequence verified by Sanger sequencing (GATC, Konstanz, Germany).

### 4.2. Cell Culturing and Transfections

Human bone osteosarcoma HOS cells (ATCC^®^ CRL-1543™), human cervical cancer HeLa cells (ATCC^®^ CCL-2) and human lung cancer A549 cells (ATCC^®^CCL-185™) were cultured in Dulbecco’s Modified Eagle Medium (DMEM) supplemented with 10% FBS, 1% glutamine, 1% antimycotic/antibiotic (Gibco, Thermo Fisher Scientific, MA, USA) at 37 °C, 5% CO_2_ and subcultured according to manufacturer’s procedure.

### 4.3. Luciferase Reporter Assay

Luciferase activity was measured with the Dual-Luciferase^®^ Reporter (DLR™) Assay System (Promega, Madison, WI, USA) according to the manufacturer’s procedure. Cells were seeded at the density 5 × 10^4^ cells/well in 24 well plates in antibiotic free growth media 24 h prior transfection. Next day, 500 ng of respective pGL3 reporter plasmid harbouring RANKL promoter (pGL3-RANKL370, pGL3-RANKL100, pGL3-RANKL183), its mutant (pGL3-RANKL370 GREMut), positive control (pGL3-SV40) and negative control (pGL3-Basic, empty vector) and together with 15 ng of pRL Renilla plasmid were transfected using 1.5 µL PolyJet™ In Vitro DNA Transfection Reagent (SignaGen Laboratories, Rockville, MD, USA) according to manufacturer’s instructions. Luciferase assays were performed 24 h after transfection. The Firefly (Firefly luciferase is encoded in pGL3 vectors) and Renilla luciferase (Renilla is encoded in pRL vector) activities were measured for each sample. Relative luciferase activity was calculated by normalizing Firefly luciferase activity with the Renilla luciferase. All the experiments were performed at least three times independently.

### 4.4. Quantitative PCR

Expression of *RANKL* was analyzed using qPCR with RNA samples obtained in gain-of-function and loss-of-function experiments. The RNA was extracted from cells and the complementary DNAs (cDNAs) were synthesized using High-Capacity cDNA Reverse Transcription kits (Thermo Fisher Scientific, Waltham, MA, USA), with gene expression analyses performed as described below. Expressions of *RANKL*, *NR3C1* were also analyzed using qPCR assays [12]. For qPCR, 5× Hot FirePol EvaGreen qPCR Mix Plus (Solis, BioDyne, Tartu, Estonia) was used, following the manufacturer recommendations, on a LightCycler 480 (Roche Diagnostics, Mannheim, Germany). Concentration of each primer in the qPCR reaction was 150 nM. Nucleotide sequences of primers are listed in Supplementary Table S1. All of the cDNA samples were diluted to the final concentration of 2.5 ng/µL. All of the samples were quantified in triplicate. Dilution series of cDNAs were prepared to create a relative standard curve, and absolute quantification of the data was performed using the second derivative maximum method (LightCycler 480, Software version 1.5; Roche Diagnostics, Mannheim, Germany). All of the data were normalized to the internal housekeeping genes of ribosomal protein, large, P0 (RPLP0) or glyceraldehyde 3-phosphate dehydrogenase (GAPDH).

### 4.5. Western Blotting

Human bone osteosarcoma cells were seeded in 10-cm^2^ dishes and transfected with empty pCMV-FLAG expression vector or pCMV-NR3C1 expression vector. The cells were lysed after 24 h and resolved on SDS-PAGE. The proteins were transferred to nitrocellulose membranes, which were later blocked in 5% milk/TTBS, and incubated overnight with the primary antibodies against FLAG (clone M2; Sigma-Aldrich, St. Louis, MO, USA). After the incubation in the primary antibodies, the membranes were washed in TTBS and incubated in the secondary antibodies bound to HRP, for 1 h. Substrate was then added and the proteins were visualized using imaging platform (Uvitec Alliance, Cambridge, UK). Antibodies against beta-actin (A2228, Sigma-Aldrich) were used for loading controls.

### 4.6. RNA Interference

siRNAs for silencing of *NR3C1* (sc-35505) and a negative control (sc-37007) were purchased from Santa Cruz Biotechnology, Dallas, TX, USA. Here, 50 pmol was transfected using Oligofectamine reagent (Invitrogen Life Technologies, Carlsbad, CA, USA). Then, 72 h after transfection, the RNA was isolated using PeqGold RNA extraction kits (VWR, Radnor, PA, USA), reverse transcribed, and subjected to q-PCR for measurements of expressions of the genes of interest. Three biological replicates were analyzed.

### 4.7. Electrophoretic Mobility Shift Assays

Electrophoretic mobility shift assays were performed using LightShift Chemiluminescent EMSA kits (Thermo Fisher Scientific, Waltham, MA, USA), according to the manufacturer instructions and as previously described [12]. 293T nuclear extracts were prepared using NE-PER Nuclear and Cytoplasmic Extraction Reagents (Thermo Fisher Scientific, Waltham, MA, USA), according to manufacturer instructions. Double-stranded biotinylated oligonucleotides (probe sequence 5′CAGCGTCGCCC**TGTT**CTTCTATTTCAGAG′3) were prepared by hybridization in a C1000 thermal cycler by stepwise 0.5 °C decreases in the temperature, ranging from 95 °C to 35 °C, over 120 cycles, each 30 s long. Control reaction was performed without nuclear extract. The specificity of the DNA-protein complexes was verified by binding with competitive non-biotinylated DNA or mutated non-biotinylated DNA. The biotinylated oligonucleotides listed in Supplementary Table S1 were used in the EMSA experiments. After the binding reactions, the samples were loaded onto electrophoretic gels and run for 1 h (100 V), then transferred to nylon membranes (30 min, 100 V), and cross-linked on a transilluminator. Biotin-labeled nuclear probes were detected with Chemiluminescent Nucleic Acid Detection Module kits (Pierce, Thermo Scientific, Wilmington, DE, USA), according to the manufacturer instructions.

### 4.8. Statistical Analysis

All experiments were performed in triplicates. Data were analysed using the GraphPad Prism 8 software (Graph Pad Software Inc., La Jolla, CA, USA). All of the data are presented as mean relative target gene expression after normalization with GAPDH expression. The results in the graphs are depicted as the means and standard error of the means of all three biological repeats. Statistical analyses were carried out by Student’s t-test. The level of significance was taken to be *p* < 0.05. In case of the luciferase assays, the results of firefly luminescence were normalized with Renilla luminescence values. The significance of the data was analysed by student t-test (two tailed) or ANOVA as implemented in Graphpad Prism 8.0. Data are presented as means with standard errors of the means.

## 5. Conclusions

In this study we demonstrate for the first time that glucocorticoid receptor can directly regulate human *TNFSF11* expression by binding to it proximal promoter region. We identified a novel glucocorticoid response element at the position +352 of the human *TNFSF11* gene and show that this site is responsible for GR dependent activation of the human *TNFSF11* promoter. Our study provides a novel link between glucocorticoid administration and onset of osteoporosis.

## Data Availability

No applicable.

## Supplementary Materials

Supplementary Materials can be found at https://www.mdpi.com/1422-0067/22/3/1054/s1. Table S1: List of primers used for plasmid construction, electrophoretic mobility shift assays and mutagenesis.

## Abbreviations

RANKL	receptor activator of nuclear factor κ B ligand
GIO	Glucocorticoid induced osteoporosis
GR	Glucocorticoid receptor
GRE	glucocorticoid responsive element
GC	glucocorticoid
NR3C1	Nuclear Receptor Subfamily 3 Group C Member
OPG	osteoprotegerin
EMSA	electrophoretic mobility shift assay
