# Supplementary material for: Glucocorticoid Receptor Regulates TNFSF11 Transcription by Binding to Glucocorticoid Responsive Element in TNFSF11 Proximal Promoter Region
Source: Int J Mol Sci. 2021 Jan 21;22(3):1054. doi: 10.3390/ijms22031054 (PMC7865994; doi:10.3390/ijms22031054)
Supplement: Supplementary file 1 [file ijms-22-01054-s001.pdf]

Supplementary table 1. List of primers used for plasmid construction, electrophoretic mobility shift assays and mutagenesis.

Primer name	Nucleotide sequence
NR3C1 probe 5' biotin f	CAGCGTCGCCCTGTTCTTCTATTTCAGAG
NR3C1 probe 5' biotin rev	CTC TGA AAT AGA AGA ACA GGG CGA CGC TG
NR3C1 probe f	CAGCGTCGCCCTGTTCTTCTATTTCAGAG
NR3C1 probe rev	CTC TGA AAT AGA AGA ACA GGG CGA CGC TG
Nr3C1mut 5' biotin f	CAGCGTCGCCCAAGACTTCTATTTCAGAG
Nr3C1mut 5' biotin rev	CTC TGA AAT AGA AGA ACA GGG CGA CGC TG
Nr3C1mut f	CAGCGTCGCCCAAGACTTCTATTTCAGAG
Nr3C1mut rev	CTC TGA AAT AGA AGA ACA GGG CGA CGC TG
RANKL 100 f	GCCAGATCTCACTAAGAGCCACAGTTCTGAATAGAGG
RANKL 100 rev	CCAAAGCTTCTTGTCTGCGGCCAACTC
RANKL370 f	GCCAGATCTCACTAAGAGCCACAGTTCTGAATAGAGG
RANKL370 rev	ACG TAA GCT TCT GCG CTC TGA AAT AGA AGA
RANKL183 f	GCCAGATCTCACTAAGAGCCACAGTTCTGAATAGAGG
RANKL183 rev	ACG TAA GCT TCC TCC GAG CCA CGC AG
GREmut f	CAGCGTCGCCCAAGACTTCTATTTCAGAG
GREmut rev	CTCTGAAATAGAAGTCTTGGGCGACGCTG
